# Premature ovarian insufficiency restoration after chemotherapy: current achievements and future prospects on its treatment or management

**DOI:** 10.1186/s13048-025-01677-4

**Published:** 2025-07-31

**Authors:** Hamideh Aboutalebi, Shayan Vafaei, Mohammad Aboutalebi, Hengameh Dortaj, Fatemeh Alipour, Alireza Ebrahimzadeh- Bideskan

**Affiliations:** 1 Department of Anatomy and Cell Biology, School of Medicine, Mashhad University of Medical Sciences, Mashhad, Iran; 2 Faculty of Medicine and Surgery, Tor Vergata University, Rome, Italy; 3 Applied Biomedical Research Center, Mashhad University of Medical Sciences, Mashhad, Iran

**Keywords:** Ovarian tissue, Chemotherapy, Infertility, Therapeutics

## Abstract

One of the important discussions in assisted reproductive technology (ART) is to maintain fertility in those who are at risk of losing their fertility for various reasons, including cancer and the use of anti-cancer therapies, hence finding a way to maintain fertility during chemotherapy, is vital. Nowadays, in addition to successfully treating patients, oncologists have also focused their attention on preserving their patients' potential of the latter to conceive. Chemotherapy-related ovarian failure, which manifests as a non-physiological form of amenorrhea, can cause dysfunction of the ovary. It is hypothesized that chemotherapeutic agents may cause DNA damage, accelerate follicular apoptosis, oxidative stress, resulting in loss of ovarian reserve function. Hence investigation on utilization of alternatives in order to maintain ovarian function and fertility in cancer survivors seems important. This review provides an update on available and potential future prospects for fertility preservation in women treated with chemotherapy.

## Key points

1. The protection of ovarian tissue against the deleterious effects of chemotherapy agents is a multifaceted challenge that necessitates a comprehensive approach.

2. Cryopreservation techniques, such as oocyte, embryo, and ovarian tissue cryopreservation, have emerged as pivotal strategies.

3. In addition to cryopreservation, the use of ovarian protection agents, such as gonadotropin-releasing hormone (GnRH) agonists, has shown promise in reducing chemotherapy-induced ovarian damage.

4. Apoptosis inhibitors represent another promising avenue for protecting ovarian tissue.

5. Physical activity, when done regularly and with proper guidance, offers significant physiological and psychological benefits for patients.

6. Mounting evidence indicates that stem cells (SCs)-derived conditioned medium (CM) has emerged as a promising new and efficient therapeutic option for various ovarian injuries.

7. Tissue engineering approaches offer a novel and promising avenue for protecting and restoring ovarian function.

## Introduction

There is no standard definition for infertility but according to the WHO, it define as being unable to get pregnant six months or one year of regular unprotected sexual intercourse [1]. Infertility is a common and worrying issue for couples and 50% of infertility cases in couples are due to female factors [2]. Infertility in women may result from multiple factors including defects in ovulation, underdevelopment of the gonads, ovarian tumors, peritoneal infections, birth defects of the structure of the reproductive system, abdominal surgery, sexually transmitted diseases, abnormal cervical mucus, weight gain, and aging. All of these can reduce the quantity and quality of oocytes and leading to temporary or permanent infertility [3, 4]. Therefore, any alteration at all different levels of the axes involved in reproduction disorders may occur and disrupt ovulation resulting in infertility [1]. One of the most important causes of women infertility is ovulation problems which is associated with various reasons including lack of ovulation, gonadal agenesis, ovarian tumor, and premature ovarian insufficiency (POI) [5].

## Chemotherapy agents and POI

POI is a clinical condition causing amenorrhoea, hypergonadotropic hypogonadism, and infertility in women under 40 years old [6] with diverse etiologies including idiopathic causes, genetic disorders, ovarian autoimmune causes, infectious agents, environmental toxins, and iatrogenic factors [7–10]. Chemotherapy is one of the most important iatrogenic causes of POI [7]. Cancer treatment damages different body organs including the ovaries, which depends on the type of treatment and the age of the treatment reception. Younger women (less than 40 years old) are at lower risk of premature ovarian infertility than older women [8, 9]. The cytotoxic mechanisms of different chemotherapeutic agents are diverse and not unequivocally understood. However, chemotherapy drugs can stop ovulation and prematurely sterilize the ovaries [10]. These drugs are divided into five groups according to their function, which include: alkylating drugs, anti-metabolic drugs, aneuploidy inducers, radiomimetics, and topoisomerase II inhibitors [11].

## Chemotherapy agents and oxidative stress

Although controlled production of reactive oxygen species (ROS) are essential for many cellular processes, overproduction generation of ROS results in oxidative stress (OS) [12]. Many of chemotherapy agents exert their therapeutic effects via oxidative mechanism, which often result in increased ROS.

Despite having positive effects of OS on enhancing anticancer and therapeutic properties of chemotherapeutic agents, excessive OS would affect normal tissue cells as well [12].

In the state of OS many macromolecules are damaged and the process of lipid peroxidation, DNA oxidation, protein oxidation, enzyme inactivation, dysfunction of various membranes, apoptosis, and necrosis occur [13–16].

Studies have shown that in steroidogenic tissues such as the ovary, ROS can be produced as a result of the separation of electron transfer from the hydroxylation of the substrate by cytochrome P450 enzymes. In the ovary, ROS play a critical role in modulating oocyte growth, triggering ovulation, regulating meiosis, and orchestrating other physiological functions. however, rise in ROS levels in conjunction with a reduction in pre-ovulatory antioxidant levels disrupts the ovulation [17]. Evidence suggests that OS contributes to the pathogenesis of multiple diseases including POl by increasing the generation of free radicals which in turn activates the apoptotic pathways within the ovarian granulosa cells [18]. In addition, reducing the activity or amount of endogenous antioxidants can be effective in exacerbating this condition. The surge of ROS caused by chemotherapy agents can overwhelm the endogenous antioxidants defense system [19]. Apoptosis and follicular atresia.

During the intrauterine period in the human ovaries, the number of primordial germ cell peaks around midgestation roughly 7 million a large number of them undergo apoptosis before birth [20].

Cell death persists after birth, resulting in an approximate 400,000 follicles remaining in both ovaries by puberty. out of the total follicular reserve present at the onset of puberty, only about 400 follicles undergo ovulation and are successfully released throughout a woman's reproductive lifespan. Over 99% of remaining ovarian follicles undergo degeneration throughout the reproductive lifespan through a physiological process known as postnatal follicular atresia. this phenomenon represents a crucial physiological mechanism of negative selection during the growth and development of ovarian follicles with pivotal role in determining the fate of ovarian follicles and fertility [21, 22]. Apoptosis is thought to have a role in mediating this process [23, 24].

**Fig. 1:**
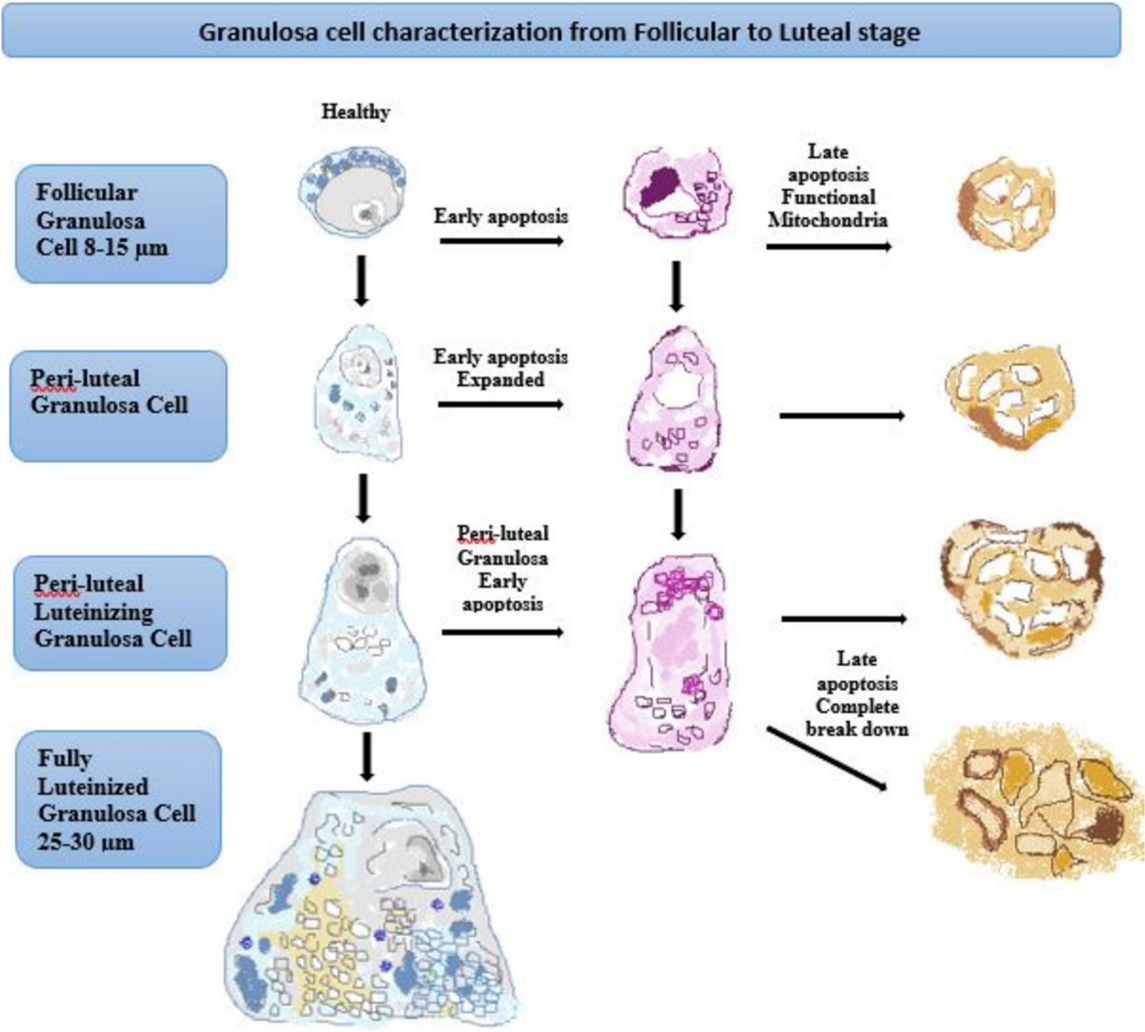
Schematic diagram of granulosa cell characteristics from follicular to luteal phase. In a stage-dependent developmental process, the granulosa cell differentiates from a compact 8–15 µm cell with a large round nucleus and relatively small cytoplasm. The cytoplasm contains mitochondria, rough endoplasmic reticulum, Golgi apparatus, lipid droplets, and many other organelles. As the granulosa cells mature, the organelles proliferate and the cytoplasm expands to acquire new steroidogenic capacity. A fully luteinized granulosa cell, 25–30 μm, contains a large volume of mitochondria, steroid-rich lipid droplets, and smooth/rough endoplasmic reticulum. This cell can directly produce progesterone. At any stage of follicular development, granulosa cells may undergo apoptosis. Early apoptosis is characterized by the destruction of the cell membrane and condensation of chromatin, which is often polarized in the nucleus with adjacent organelles. Early apoptosis in a granulosa cell with an enlarged cytoplasm is similarly modified by an increased volume of organelles surrounding a collapsing nucleus. The final stages of apoptosis end with organelles collapsing into vesicles and expelling apoptotic bodies that may contain nuclear material

Growth factors such as EGF, TGF-β, IGF-1, and FGF as along with gonadotropins and IL-1β have been recognized as important follicule survival factors. In addition, estrogen and inhibin are known to inhibit follicular atresia, while androgens, activin, IL-6, TNF-α, and FasL induce atresia [25–28]. Research finding have showed that follicular atresia initially begins with the apoptosis of follicular granulosa cells, followed by the subsequent death of other follicular components [29]. The hallmark morphological features of apoptosis are cytoplasm blebbing, chromatin condensation, nuclear and cell fragmentation, and the formation of apoptotic bodies, which first appears in granulosa cells and subsequently extends in the theca cell layer (Fig. 1) [30].

## Apoptotic pathways in granulosa cells

Apoptosis in cells occurs through two distinct pathways: the external and internal pathways, both of which will be elaborated upon in the following section.

**Fig. 2:**
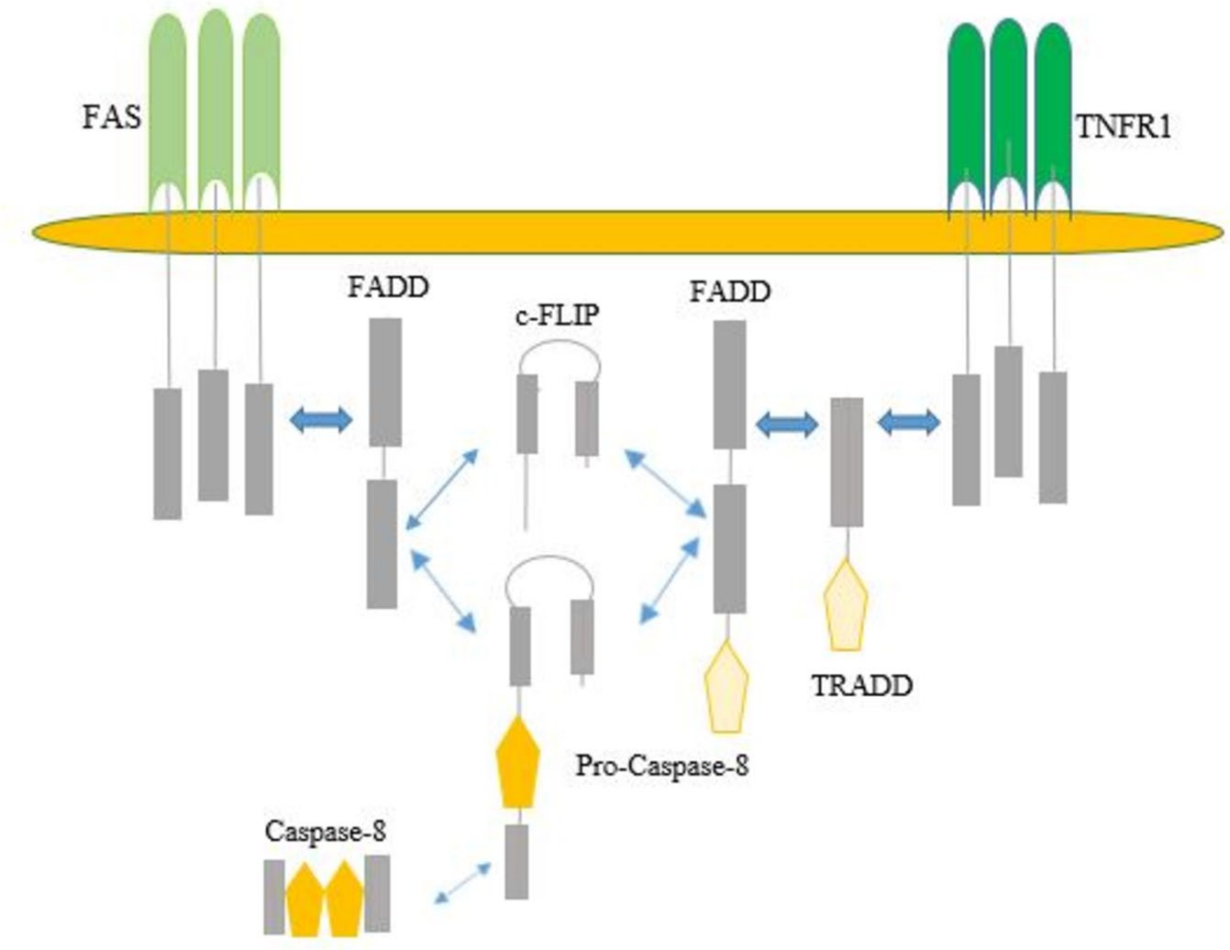
Surface receptor TNFR1, and its associated apoptosis pathway. In this pathway, when the TNF-α ligand binds to the TNF-R1 receptor, the TRADD molecule is activated inside the cell and through DD reaction to the inner second of the TNF-R1 receptor. It is connected and through the DED-DED reaction on procaspase 8 or its active form, it triggers the caspase cascade, which then activates caspases 6 and 7 respectively, and then caspase 3 is activated, which is the final executor of cell death and apoptosis

**Fig. 3:**
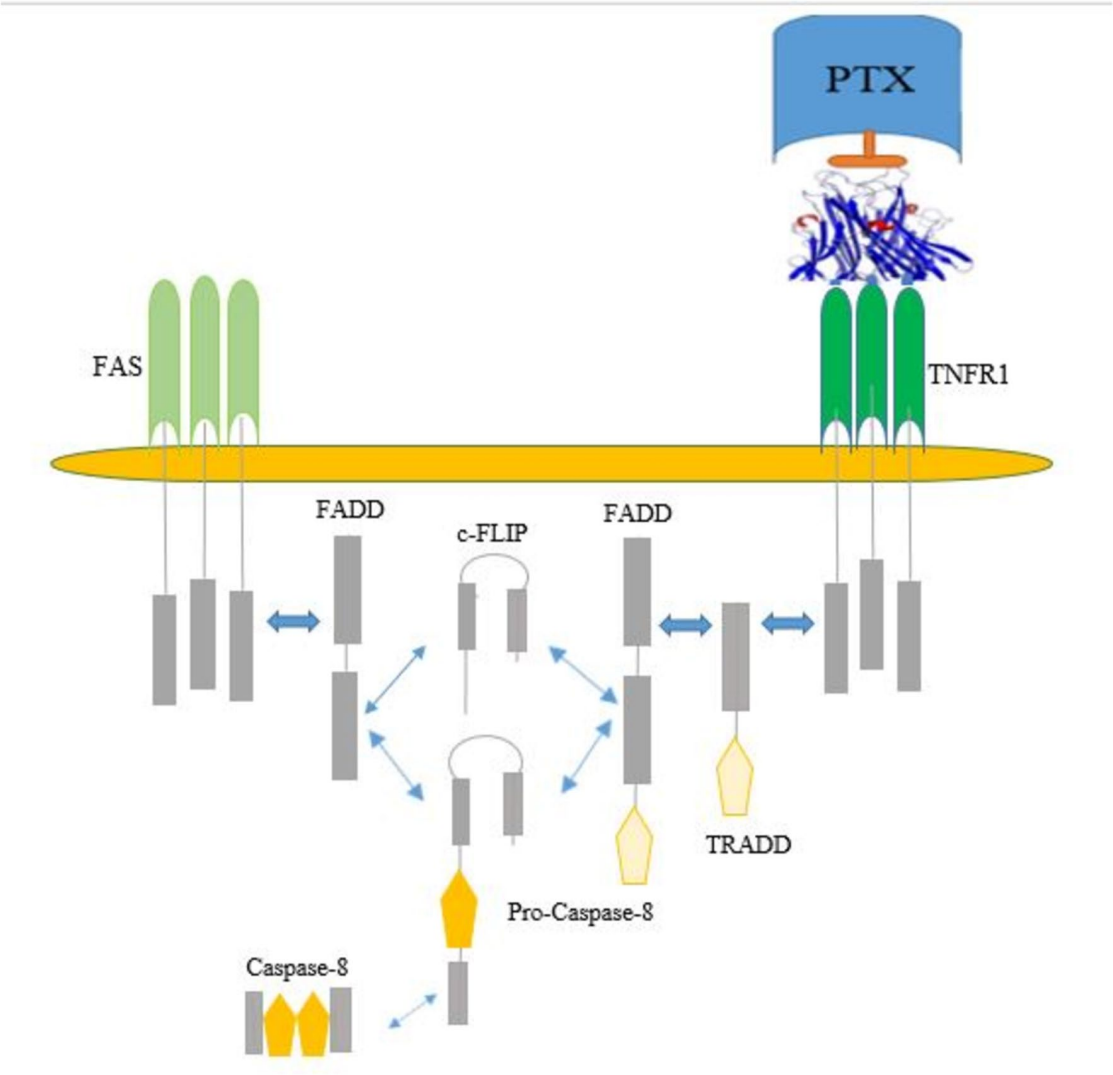
The above picture shows the mitochondrial pathway of apoptosis. Apoptosis is induced through the P53 that causes the activation of BAX, inhibition of BCL2, and the release of cytochrome C from mitochondria. Cytochrome C activates caspase 9, which then activates caspases 6 and 7 and finally caspase 3. After all, apoptosis occurs

- 1- The external pathway, also known as the death receptor- mediated pathway, is initiated through death receptors located on the plasma membrane of most cells. The most important death receptors are the tumor necrosis factor (TNF) receptor superfamily, which include TNF-R1, FASR receptors, and so on. When these receptors are activated by their respective ligands, such as TNF-α or Fas L, they trigger the activation of caspases, ultimately leading to apoptosis (Fig. 2) [31].
- 2- Internal pathway or mitochondrial pathway is activated in response to intracellular signals in which increase the permeability of the mitochondrial outer membrane, resulting in the release of pro-apoptotic molecules such as cytochrome C from the mitochondria into the cytoplasm. Cytochrome C together with adapter proteins such as Apaf-1 activates caspase 9, which further activates executive caspases such as caspase 6 and 7 and induces apoptosis. Several regulatory genes such as p53, Bax, and Bcl-2 are involved in the modulation of this pathway of apoptosis (Fig. 3) [32–35].

Ovarian tissue response to free radicals caused by oxidative stress through two different defense mechanisms:

- 1- Enzymatic antioxidant system, which includes key enzymes such as glutathione peroxidase (GSH), superoxide dismutase (SOD), and catalase (CAT) [36].
- 2- Non-enzymatic antioxidant system, consisting of some of the most important molecules like vitamins E, A, C, and carotenoids [37]. In addition to the aforementioned systems, the cells employ anti-apoptotic proteins, such as members of Bcl2 family to inhibit the progress of apoptosis in different stages of cell death. These proteins directly inhibit the release and activation of mitochondrial pro-apoptotic factors like cytochrome C. Furthermore, they suppress the activity of initiator caspases and regulate the executive caspases action [35].

Studies show that in primary follicles (pre-antral), the survival rate largely influenced by survival factors secreted by the ovum [38]. At this stage of follicular development, several factors can contribute to germ cells death including insufficient access to survival factors like IGF-1 and GDF-9, genetic defects, or exposure to chemotherapeutic agents. Studies also show that the granulosa cell layer may receive cell survival signals from germ cells or the adjacent theca layer [35].

In the antral follicles, two distinct patterns of cell death have been identified: 1- antral atresia, in which apoptotic cell death initially occurs in the granulosa cells adjacent to the antrum, potentially due to factors from granulosa layer or even the ovary. 2- basal atresia, in which cell death begins in granulosa cells near the basement membrane, possibly triggered by external factors. Thus, multiple pathways determine cell death or survival. however, the thing that plays an important role in determining the fate of ovarian germ and somatic cells is the complex interplay between endocrine, paracrine, and autocrine factors, as well as the interactions between tumor suppressor genes, survival genes, and death genes [21, 35].

## Update on therapeutic strategies for POI during chemotherapy

### Platelet-rich plasma

**Fig. 4:**
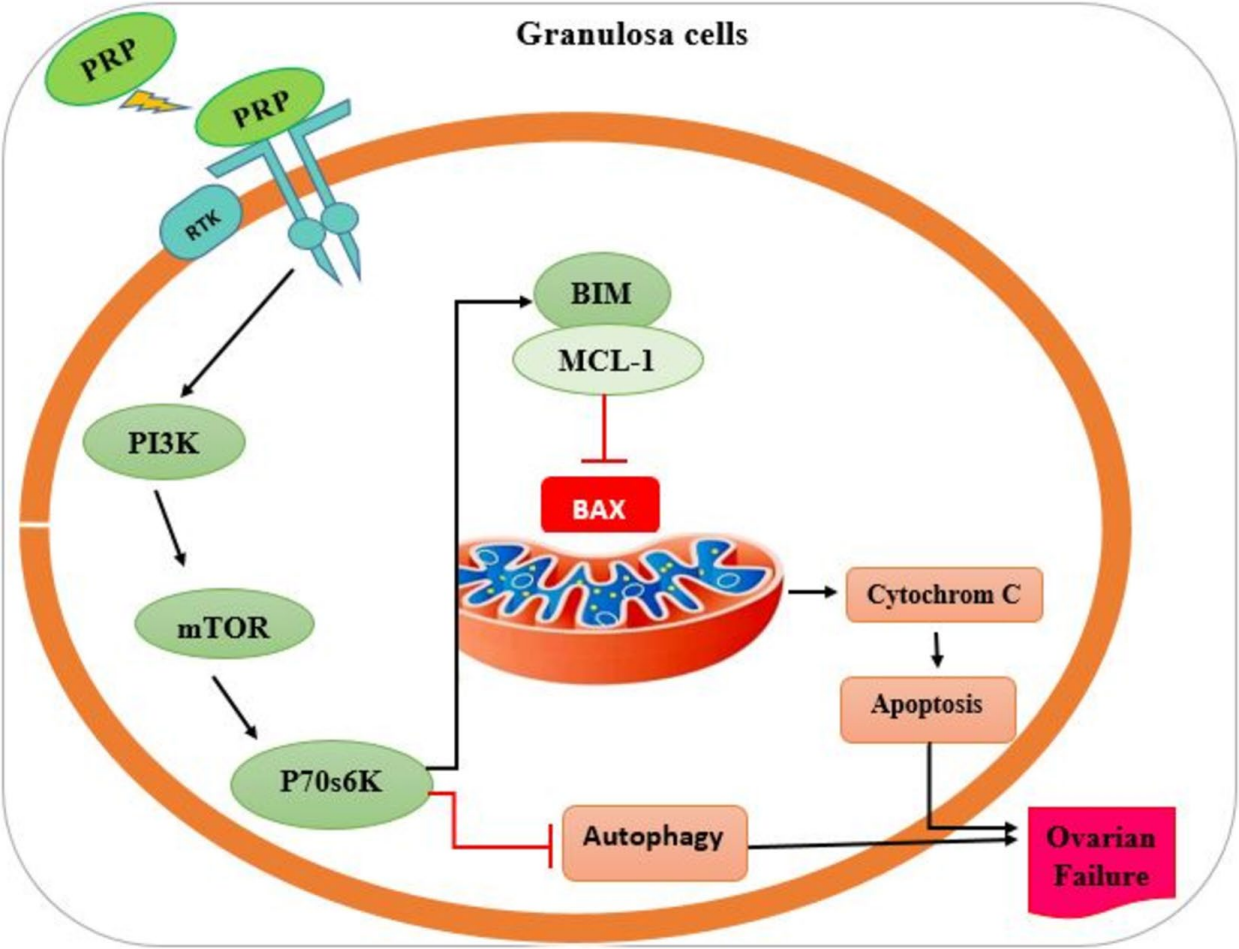
PRP and suppression of the mitochondrial pathway of apoptosis. Through the PI3 K-mTOR pathway, PRP activates P70 s6 K enzymes, then BIM and MCL-1, which ultimately inhibits BAX and prevents the release of cytochrome C and, as a result, cell apoptosis

Platelet-rich plasma (PRP) is an autologous blood-derived biological product characterized by a high concentration of platelet. it contains a broad spectrum of growth factors such as CTGF, VEGF, FGF, IGF, EGF, PDGF, TGF-B, and IL-8. These compounds have the potential to increase tissue repair through cell migration, proliferation, and differentiation. PRP has been shown to stimulate stem cells, promoting their proliferation and differentiation [39]. Moreover, PRP is rich in growth factors that suppress BAX through activation of PI3 K-Akt-mTOR signaling pathway, thereby inhibiting mitochondrial apoptotic pathway. This involve the prevention of cytochrome C release from mitochondria, which is a critical event in the intrinsic pathway of apoptosis (Fig. 4) [40].

Since the 1970 s, researchers have concluded that bioactive agents, such as growth factors, are highly effective in repairing damaged tissues in the body. as a result, PRP has emerged as a practical approach for extracting growth factors [41]. PRP products consist of platelets concentrates, platelet gels, and concentrated growth factors, which have been evaluated through both in vivo and in vitro studies. PRP typically contains varying amounts of plasma, red blood cells, white blood cells, and platelets, with an accepted standard indicating that fortified platelets should have at least fivefold higher than that of the whole blood [42]. Studies have shown a positive correlation between platelet concentration and human mesenchymal cell proliferation, fibroblast proliferation, and type 1 collagen production [39]. In recent years, PRP has been used to regenerate soft and hard tissues in the fields of orthopedics, dentistry, maxillofacial surgery, urology, metabolic bone diseases, degenerative joint disease, bone fracture repair, and treatment of wrinkles caused by enlargement. Furthermore, PRP has been shown promising results in hair loss repair, and baldness treatment at the early stages [39, 40, 43–45].

Beyond being rich in growth factors, PRP has gained popularity in reproductive context. It is now being explored as a novel and alternative therapeutic strategy to enhance follicular growth, maturation, and appears to effectively increase ovarian reserve [46, 47]. Emerging evidence Studies have reported suggests that the growth factors present in PRP may contribute to increase blood estrogen levels, thereby stimulating ovulation and enhancing fertility outcomes [36].

### Tumor necrosis factor alpha

Tumor necrosis factor alpha (TNF-α) is a pleiotropic adipokine and pro-inflammatory cytokine that plays a central role in mediating systemic inflammatory responses, particularly during the acute phase of inflammation. This inflammatory factor is mainly secreted by macrophages, lymphocytes, mast cells, adipocytes, cardiac myocytes, endothelial cells, and neurons are produced [48, 49]. Within the ovary, various cell types including granulosa cells and tissue-resident macrophages produce TNF-α, with its biological impact depending on both concentration and the target cell [50]. In ovarian environment, TNF-α plays a role in follicular growth, ovulation, and luteolysis, while also being implicated in follicular atresia and pharmacological apoptosis in granulosa cells under OS conditions [51]. Today, pharmacological treatment is done by inhibiting TNF-α in diseases such as rheumatoid arthritis, psoriasis, asthma, and irritable bowel. The increase of TNF-α factor in the inflammatory response in turn causes clinical problems with autoimmune disorders. Phosphorylation events in the TNF-α signal or message play an important role in modulating cellular responses to regulate cell death or life [52, 53].

### Pentoxifylline

**Fig. 5:**
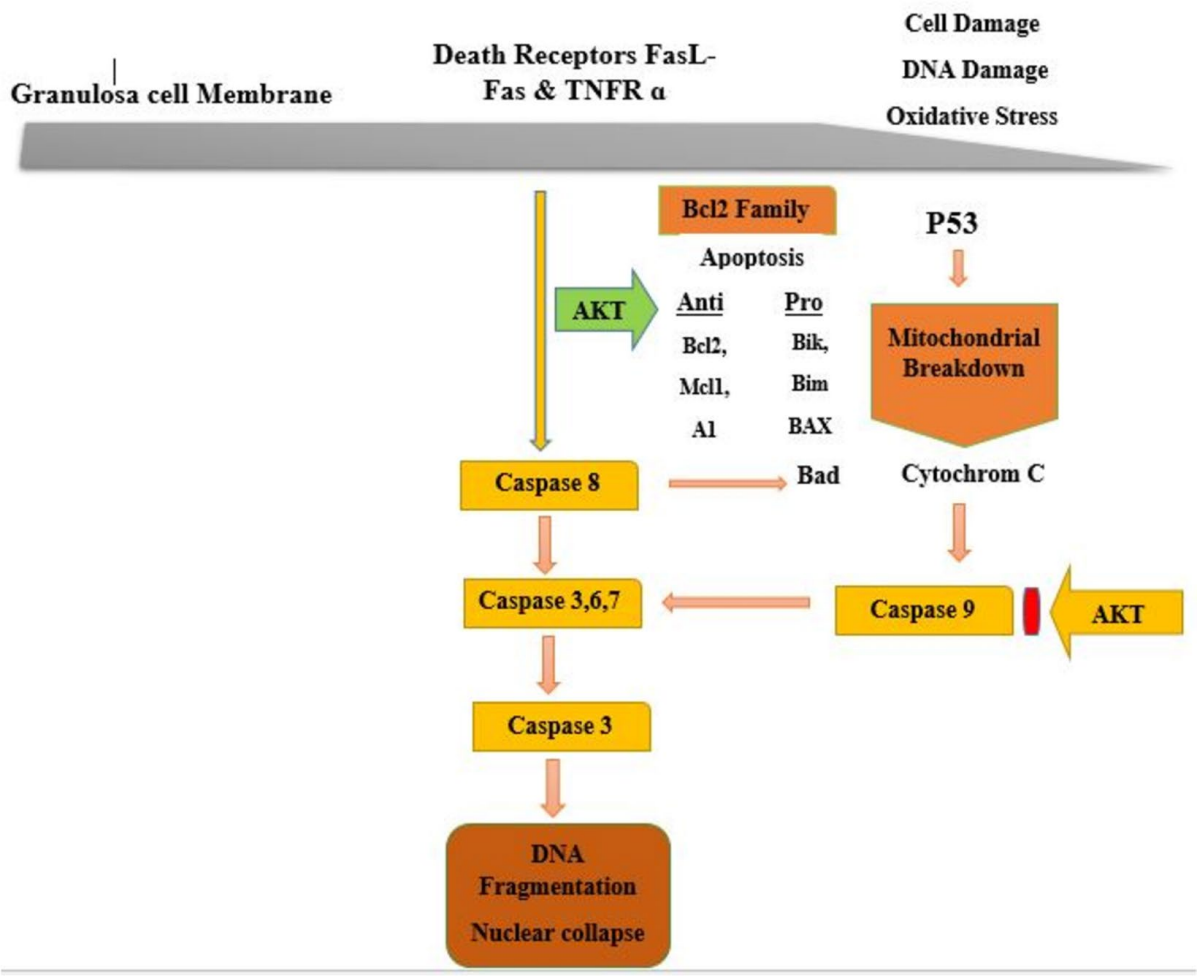
PTX and apoptosis receptor pathway suppress. PTX blocks the apoptosis process from the cell receptor pathway by α-TNF inhibition

Pentoxifylline (PTX) is a synthetic derivative of methylxanthine, is widely used in the treatment of peripheral vascular diseases. while it is often categorized as a vasodilator, its primary activity is to reduce blood viscosity, which is achieved probably through improved erythrocyte deformability, along with the inhibition of platelet adhesion and aggregation. This drug increases blood flow in ischemic tissues, leading to enhanced tissue oxygenation in patients with peripheral vascular diseases. beyond its hemorheological benefits, PTX exerts anti-inflammatory effects by downregulating cytokine production, particularly the TNF-α (Fig. 5) [54].

As a non-competitive inhibitor of phosphodiesterases, PTX increases intracellular cAMP and activates PKA, inhibits α-TNF factor, and reduces the production of leukotrienes. Consequently, PTX exhibits anti-inflammatory and immunomodulatory effects, enhancing innate immune function [55–57].

Mechanistically, PTX functions as an adenosine 2 receptor antagonist, promoting intracellular cAMP accumulation in endothelial cells, red blood cells, and surrounding tissues thereby making red blood cells more flexible to absorb oxygen and increase oxygen delivery to the tissue, by inhibiting phosphodiesterase [58, 59]. Moreover, by inhibiting the production of TNF-α and interferon-gamma induces T-helper 2 (Th2)to produce cytokines and suppress Th1-mediated responses that are responsible for inflammatory and autoimmune responses [55]. Additionally, its ability to e neutralize ROS endows PTX with antioxidant properties although it does not appear to significantly impact GSH reserves [58, 59].

### MicroRNAs

MicroRNAs (miRNAs) are highly constitute a conserved class of small non-coding RNAs that have recently emerged as therapeutic agents. However, miRNA may operate as a double-edged sword. miRNA-based therapeutics have been recently reported clinical trials, especially in the field of oncology [60]. Recent evidence highlights that miRNAs have a key regulators of follicular growth, atresia, and steroidogenesis [61, 62]. Given their regulatory capacity, elucidating the role of miRNAs in response to gonadotoxic agents, including chemotherapeutic drugs, may offer novel fertility-preserving strategies. Targeting specific miRNAs could mitigate follicular damage and preserve reproductive potential during cancer treatments [63].

### Stem Cell-based therapy

Stem cells (SCs) are defined as a group of cells that have ability to self-renew and differentiation into various cell lines [64, 65]. SCs can be isolated from embryonic or adult tissues, and are generally categorized into embryonic stem cells (ESCs), adult stem cells (ASCs) mesenchymal stem cells (MSCs), and induced pluripotent stem cells (iPSCs) [66]. Additionally, a unique ovarian-resident population known as very small embryonic-like stem cells (VSELs) which can differentiate into oocyte-like cells [67]. Accumulating evidence suggests that SCs-based therapies have shown efficacy in treating female infertility [68]. Today, stem cell therapy (SCT) is a popular treatment plan for various diseases including degenerative ones [69, 70]. SCT has been also used in purpose to regenerate oocytes and ovarian functions [71]. the therapeutic effects of SCT not only through the direct differentiation into germ cells but also via exosome secretion and paracrine signaling function can modulate inflammation and apoptosis [72, 73]. Furthermore, SCT therapy has demonstrated a positive effect on female infertility disorders such as POI, not only in animal models [74, 75], but also in human clinical trials [76, 77].

### Conditioned medium

As mentioned above, SCs secret paracrine factors known as conditioned medium (CM) which contributes to its therapeutic effect. this medium contains a diverse array of bioactive molecules such as Hepatocyte growth factor (HGF), Vascular-endothelial cell growth factor (VEGF), Insulin-like growth factor (IGF), Interleukins (e.g., IL-8, IL-10, IL-11, IL-15) importantly, CM is also rich in exosomes and microvesicles, which serves as vehicles for the delivery of cytokines, growth factors, and nucleic acids to target cells, thereby modulating cellular behavior and promoting tissue repair [71, 78–81]. Several studies have highlighted the anti-inflammatory, anti-apoptotic, and tissue-protective properties of CM derived from different SCs sources, particularly in the context of female reproductive health [82, 83]. Notably, an in vitro study in 2019 showed that cumulus cell CM can meaningfully enhance germ cell maturation and upregulates the expression of germ cell-specific markers, indicating its potential role in folliculogenesis [84]. Additionally, findings from Jiao et al. (2020) underscore that CM, irrespective to its cellular origin, may exert a protective effect against ovarian aging [85], offering a cell-free therapeutic alternative for infertility management.

## Potential novel strategies to manage or treat POI

### Physical activity and health promotion

Regular physical activity is increasingly recognized as a valuable non-phamacological intervention for mitigating the adverse effects of cancer treatment [86]. Oncology nurses, who frequently interact with chemotherapy patients, are ideally positioned to educate and empower these individuals to exercise appropriately and consistently. The potential to improve health outcomes through exercise intervention is undeniable. Raising awareness and providing practical exercise recommendations to patients undergoing chemotherapy is vital [87].

#### Physiological and Psychological Benefits

Lis Adamsen's study in 2009 showed that regular physical activity can lead to numerous physiological benefits, such as improved cardiovascular health, enhanced muscular strength, and increased overall endurance in patients undergoing chemotherapy. Beyond these physiological gains, exercise can help counteract common treatment-related side effects, including fatigue, nausea, and muscle weakness [88]. Furthermore, exercise contributes to maintaining a healthy weight and improving overall physical function, which is crucial for patients who may experience muscle loss and weight fluctuations due to cytotoxic therapies [84–86, 89]. Another study in 2015 showed that psychologically, exercise reduce symptoms of anxiety and depression, improve mood, and enhance self-esteem [90]. For cancer patients, these psychological benefits are particularly important, as they often face emotional challenges related to their diagnosis and treatment [91]. Additionally, interventions such as supervised exercise training program (SETP) have demonstrated efficacy in preserving health-related quality of life (HRQoL) and maintaining functional capacity of women with cancer undergoing chemotherapy [90]. The supervised nature of this program ensures patients safety while maximizing the therapeutic benefits of physical activity in cancer care [92].

#### Examples of exercises

Incorporating tailored exercise into the daily routine of cancer patients undergoing chemotherapy can be highly beneficial. Here are some examples of exercises that can be particularly effective:

- 1. Walking: A low-impact activity offers a flexible option that can be adapted to the patient’s energy levels. Starting with short walks and gradually increasing the duration and intensity can help build stamina and improve cardiovascular health without overwhelming the patient [93, 94].
- 2. Gentle Yoga: This form of exercise emphesize flexibility, postural control, and stress regulation. Numerous clinical trials have shown that yoga can significantly improve mental well-being, reduce anxiety, and foster relaxation in patients [95].
- 3. Resistance Training: Using light weights or resistance bands has been widely endorsed for maintaining muscle strength and bone density. It's important that patients perform these exercises with proper form, possibly under the supervision of a trained professional, to avoid injury [86, 96].
- 4. Swimming or Aquatic Exercises: These activities are gentle on the joints and the buoyancy of water reduces stress on the body, making it an ideal exercise option for those experiencing joint pain or fatigue [97, 98].
- 5. Tai Chi or Qigong: These practices are forms of meditative therapies, emphasize controlled, mindful motions along with deep breathing which can enhance balance, reduce stress, and promote relaxation. Those interventions are particularly beneficial for improving mental focus and reducing anxiety [99].

Collectively, these findings suggest that oncology nurses should actively promote exercise as a health-enhancing intervention during chemotherapy treatment. By educating patients about the benefits of exercise and providing them with practical, personalized recommendations, oncology nurses can play a pivotal role in helping patients incorporate physical activity into their treatment plan. Emphasizing how exercise contributes to well-being can encourage cancer patients to adopt exercise as a health-promoting activity that aids their recovery, ultimately improving their quality of life [100, 101].

Furthermore, based on the previous studies, there is a positive relation between regular moderate physical activity and ovarian reserve in overweight and obese women [102–105]. It was suggested that sedentary personal lifestyles may elevate the risk of developing POI [106]. Importantly, physical activity has been recognized as a supportive modality within the framework of complementary and alternative medicine for managing POI [107] symptoms and potentially improving reproductive outcomes.

### Bioengineering approaches

Tissue engineering represents a cutting-edge biotechnological approach that integrates cell biology and materials science to fabricate tissues or organs, either in vitro or in vivo, with the aim of restoring or replacing the functionality of damaged or diseased tissues and organs.

Bioengineering strategies for addressing ovarian failure post-chemotherapy focus on innovative techniques such as stem cell therapy and biomaterials to restore ovarian function. These approaches aim to mitigate damage caused by chemotherapy, enhance cell survival, and promote tissue regeneration, ultimately improving fertility outcomes for affected women.

Reproductive tissue engineering (RTE) is a promising field that uses techniques from creating vital organs to develop biomaterials that can regenerate ovarian tissue, including follicles [108]. In the absence of tissue engineering strategies, factors such as inflammation, apoptosis, and ischemia at the transplant site can further compromise cell viability within the target organ. Moreover, the rapid dispersal of injected stem cells into adjacent tissues can hinder their localization and engraftment in the intended site. A breakthrough is the successful creation of an extracellular matrix (ECM) that supports oocyte development and maintenance. To be suitable for use in biological systems, biomaterials must meet strict requirements, including being biocompatible, non-toxic, degradable, breathable, and allowing cells to attach and move freely [109, 110].

Biomaterials are pivotal in the realm of regenerative medicine, serving as essential components in tissue engineering. Ideal biomaterials exhibit key characteristics, including biocompatibility, the ability to enhance cell function and viability, and the promotion of favorable cellular interactions. They should also possess attributes such as effective passive and active targeting, stability, biodegradability, high drug loading capacity, and controlled drug release. An optimal biomaterial must be non-toxic, biocompatible, biodegradable, and bioabsorbable, facilitating the regeneration of new cells and tissues without eliciting inflammatory responses. Generally, biomaterials can be classified into three categories based on their origin: natural biomaterials, synthetic biomaterials, and composite biomaterials.

Tissue-engineered scaffolds can be seeded with stem cells or ovarian cells, promoting their integration into the existing ovarian tissue. This integration is vital for restoring normal ovarian function and hormone production.

Physical properties are imperative for biomaterials'bioactivity besides biological characteristics. These physical properties include malleability, structural integrity, porosity, compartmentalization, electrical properties, and interactions between polymer molecules [111]. By balancing these factors, researchers in reproductive tissue engineering aim to develop biomaterials that can support the growth and maturation of ovarian follicles, ultimately improving fertility treatments and reproductive health [112].

Multidisciplinary approaches and advantages in bio-assembly methods are the core of bio- fractionation techniques for printing transplantable tissues. By encouraging cell-to-cell and tissue-level organization through interactions with extracellular matrix components, the engineered model is expected to simulate grafts, recapitulating the structure and complexity of native tissue. Studies have consistently shown that three-dimensional (3D) culture systems outperform two-dimensional (2D) conventional systems in accurately modeling the interactions between cells and their ECM environment, resulting in data with higher reproducibility [113].

Natural biopolymers are used in tissue engineering due to their unique properties and ability to retain cells'natural behavior. Synthetic polymers like PEG and poly-e-caprolactone have also been explored as alternatives, offering biocompatibility and potential for modification. Both types of polymers can reconstruct ovarian architecture and cell repopulation, but they can still perfectly mimic the ECM, which is crucial for maintaining tissue structure and function [114].

A breakthrough discovery has revealed that hyaluronic acid, a naturally occurring substance, can effectively slow the growth of ovarian cancer cells, paving the way for innovative cancer treatments.

–Drug delivery systems (DDS) using biomaterials

In ovarian failure after chemotherapy focus on targeted and localized delivery of therapeutic agents to minimize systemic side effects and enhance treatment efficacy. These systems can utilize biomaterials to create scaffolds that support ovarian tissue regeneration and improve the delivery of stem cells or growth factors, promoting ovarian function restoration.

Tissue engineering plays a crucial role by providing a framework for cell attachment and growth, facilitating the integration of transplanted cells into the ovarian microenvironment. This approach aims to restore hormonal balance and improve fertility outcomes for women affected by chemotherapy-induced ovarian damage.

Drug delivery systems are designed to deliver therapeutic agents directly to the ovaries, minimizing systemic exposure and potential side effects. This targeted approach is essential for enhancing the effectiveness of treatments aimed at restoring ovarian function [109, 110].

#### Organ-on-a-chip system (microfluidic platform)

A novel approach to studying tissue microenvironments is the development of organ-on-a-chip technology, which miniaturizes the characteristics of a specific organ [115]. Organ-on-a-chip platforms, which combine biomimetic tissues with microfluidic systems, offer a promising alternative for investigating the ovarian microenvironment. Microfluidic systems can be integrated with tissue engineering approaches to create more sophisticated models of ovarian tissue. This can involve the use of hydrogels or scaffolds within the microfluidic channels to support cell growth and mimic the ovarian microenvironment [116].

Microfluidic technology offers a groundbreaking approach to ovarian cancer diagnosis and treatment, enabling the efficient capture and isolation of cancer cells from bodily fluids such as blood or ascites [117]. A microfluidic platform is particularly well-suited for drug testing and personalized therapy in situations where tumor material is scarce, such as after obtaining biopsy samples through fine-needle aspiration. By manipulating cells using microfluidic devices, scientists can uncover novel therapeutic strategies to target and eradicate ovarian cancer at its root [118].

- Reduced Sample Volume: Microfluidic devices require significantly less biological material compared to traditional methods, making them ideal for studies involving limited samples, such as those from patients undergoing chemotherapy.
- Enhanced Control: The ability to manipulate fluid flow and chemical gradients within microfluidic channels allows for a more nuanced understanding of how different factors influence ovarian cell behavior.
- Real-Time Analysis: Many microfluidic systems can facilitate real-time monitoring of cellular responses, providing immediate feedback on the effects of treatments and enabling dynamic studies of ovarian function.
- Personalized Medicine: Microfluidic technologies hold the potential for personalized approaches to treating ovarian failure, allowing for the testing of individual responses to various therapies based on patient-specific factors

### Current limitations

Although based on the available literature regular physical activity has positive influence on POI management by different etiology, we could not find studies demonstrating a direct correlation between physical activity and improvement of the iatrogenic ovarian failure. Thus, this represents one limitation of the current review.

Despite the promising advancements in the restoration of premature ovarian insufficiency (POI) following chemotherapy, several limitations persist that warrant consideration.

1. Variability in Patient Response: The heterogeneity in patient responses to various treatment modalities poses a significant challenge. Factors such as age, genetic predisposition, the type of chemotherapy received, and pre-existing ovarian reserve can influence treatment outcomes, making it difficult to establish standardized protocols.
2. Limited Understanding of Mechanisms: While various therapeutic strategies, including stem cell therapy and biomaterial applications, have shown potential, the underlying biological mechanisms driving ovarian restoration remain inadequately understood. This knowledge gap hinders the optimization of these approaches and the development of targeted therapies.
3. Long-Term Efficacy and Safety: Many of the current treatment strategies lack comprehensive long-term data regarding their efficacy and safety. The potential risks associated with stem cell therapies, hormonal treatments, and tissue engineering approaches need thorough investigation to ensure that they do not lead to adverse effects, such as tumorigenesis or other complications.
4. Technical Challenges in Implementation: The translation of laboratory findings into clinical practice is often impeded by technical challenges. For instance, the scalability of tissue engineering techniques and the reproducibility of microfluidic systems for drug delivery require further refinement before they can be widely adopted in clinical settings.
5. Ethical Considerations: The use of stem cells, particularly those derived from embryonic sources, raises ethical concerns that may limit their acceptance and application in clinical practice. Addressing these ethical issues is crucial for the advancement of regenerative therapies.
6. Economic Factors: The cost associated with advanced therapeutic strategies, including personalized medicine approaches and cutting-edge technologies, may restrict access for many patients. Economic considerations must be addressed to ensure equitable treatment options for all individuals affected by POI.
7. Regulatory Hurdles: The regulatory landscape for new therapies, particularly those involving genetic manipulation or novel biomaterials, can be complex and time-consuming. Navigating these regulatory pathways is essential for the timely introduction of innovative treatments into clinical practice.

## Conclusion

The protection of ovarian tissue against the deleterious effects of chemotherapy agents remains a multifaceted challenge that necessitates a comprehensive approach. Oocyte, embryo, and ovarian tissue cryopreservation have emerged as pivotal strategies for further fertility restoration. While oocyte and embryo cryopreservation are well-established, ovarian tissue cryopreservation has recently gained increased attention due to its ability to preserve a larger pool of follicles and restore both fertility and endocrine function following transplantation. Nonetheless, the variability in success rates and long-term outcomes of these techniques vary highlights the need for continued research to optimize theses protocols and improve patient outcomes.

Beyond cryopreservation, the use of ovarian protection agents, such as gonadotropin-releasing hormone (GnRH) agonists, has shown potential in mitigating chemotherapy-induced ovarian damage. These agents act temporarily suppressing ovarian function, thereby reducing the susceptibility of ovarian follicles to the cytotoxic effects of chemotherapy agents. Clinical studies have demonstrated varying degrees of efficacy, with some showing significant preservation of ovarian function and others indicating limited benefits. The variability in outcomes underscores the need for personalized treatment plans and further investigation into the mechanisms underlying the protective effects of these agents.

Apoptosis inhibitors represent another promising avenue for protecting ovarian tissue. Chemotherapy agents induce ovarian damage primarily through the activation of apoptotic pathways, leading to follicular atresia and loss of ovarian reserve. Preclinical studies have shown that apoptosis inhibitors can significantly reduce follicular loss and preserve ovarian function in animal models. However, translating these findings into clinical practice requires rigorous testing to ensure safety and efficacy in humans. Continued advancements in this area hold the potential to enhance the repertoire of strategies available for ovarian protection, ultimately improving the quality of life for female cancer survivors.

Mounting evidence indicates that SCs-derived CM has emerged as a promising new and efficient therapeutic option for various ovarian injuries. Further studies to elucidate the bench-to-bedside gap of stem cell therapy in the clinic and identification of the key effective components of SCs secretome underlie a new paradigm in cell-free therapeutic strategies for ovarian insufficiency.

Tissue engineering approaches has also gained a novel and promising avenue for protecting and restoring ovarian function. By employing biomaterials and scaffold-based systems, researchers have developed supportive microenvironments that mimic the natural ovarian tissue architecture. These engineered tissues can be used to encapsulate and protect ovarian follicles during chemotherapy, reducing the direct exposure to cytotoxic agents. Additionally, tissue-engineered constructs can facilitate the transplantation of cryopreserved ovarian tissue, enhancing graft survival and function. Preclinical studies have shown that these approaches can significantly improve follicular survival and restore endocrine function. Continued advancements in tissue engineering hold the potential to revolutionize ovarian protection strategies, ultimately improving the quality of life for female cancer survivors.

## Data Availability

No datasets were generated or analysed during the current study.

## Abbreviations

POI: Premature ovarian insufficiency
PM: Phosphoramid
ROS: Reactive oxygen species
TNF: Tumor necrosis factor
PRP: Platelet-rich plasma
TNF-α: Tumor necrosis factor alpha
PTX: Pentoxifylline
miRNAs: MicroRNAs
SCs: Stem cells
ESCS: Embryonic stem cells
ASCs: Adult stem cells
MSCS: Mesenchymal stem cells
iPSCs: Induced pluripotent stem cells
VSCS: Very small embryonic-like stem cells
SCT: Stem cell therapy
HGF: Hepatocyte growth factor
VEGF: Vascular-endothelial cell growth factor
IGF: Insulin-like growth factor
IL: Interleukin
CM: Conditioned medium
SETP: Supervised exercise training program
HRQoL: Health-related quality of life
RTE: Reproductive tissue engineering
ECM: Extracellular matrix
3D: Three-dimensional
2D: Two-dimensional
